# A review on the immunomodulatory properties of functional nutraceuticals as dietary interventions for children to combat COVID-19 related infections

**DOI:** 10.1186/s43014-023-00133-4

**Published:** 2023-04-06

**Authors:** Swati Soni, Kuppusamy Alagesan Paari

**Affiliations:** Department of Life Sciences, CHRIST (Deemed to be) University, Central Campus, Hosur Road, Bangalore, Karnataka 560029 India

**Keywords:** Functional food, Infant food, COVID 19, Antiviral, Food additives

## Abstract

COVID-19 is a significant threat to humanity in the present day due to the rapid increase in the number of infections worldwide. While most children may be spared of the direct mortality effects of the disease, those with weak immune systems are prone to adverse effects. Child mortality increases due to the stress caused to the health care system that disrupts essential health care needs such as immunisation and antenatal care. The use of functional foods (FF) aids in disease-prevention as they are known to have protective effects against COVID-19 by boosting children’s cellular and humoral immunity. Plant components such as glycyrrhizin, epigallocatechin gallate, allicin, and fucoidan exhibit antiviral properties against various viruses, including SARS-CoV 2. Microbial foods that are made of probiotics, can enhance immunity against various respiratory viruses. Food enriched with additives such as lactoferrin, piperine, and zinc can boost immunity against COVID-19. With proper definitive drug therapy not available for treating COVID-19 and most of the disease management tools rely on symptoms and non-specific supportive care, developing a functional paediatric formulation will prevent further deterioration in infant health. It is wise to investigate the toxicological aspects of Functional Foods components especially when formulating for children. The safe limits of ingredients should be strictly followed during FFs formulation. Stronger regulations with advanced analytical techniques can help to formulate functional foods into the mainstream in child nutraceuticals. The purpose of this review is to compile collective information on the functional nutraceuticals specifically for infants and children up to the age of 10 years that could confer immunity against COVID-19 and other related viruses.

**Graphical Abstract:**

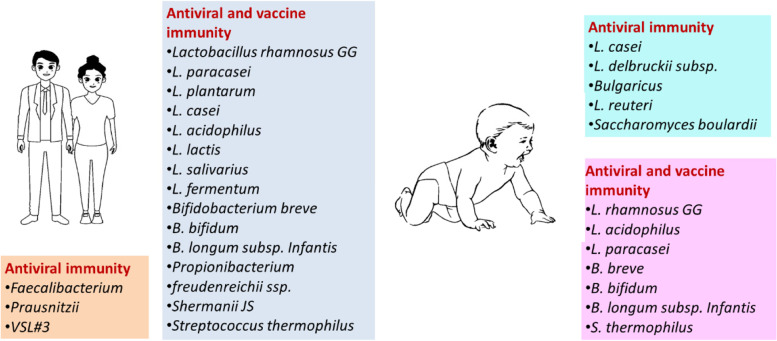

## Introduction

The causative agent of Severe Acute Respiratory Syndrome Coronavirus 2 (SARS-CoV-2) also known as “Coronavirus Disease 2019” (COVID-19) made its presence felt in the latter part of 2019 to the global scientific community. COVID-19 spreads via droplets expelled during coughing, sneezing, or talking and the symptoms of the disease can range from mild to life-threatening (Araf et al. 2021; Keni et al. 2020). As of June 2022, 53.83 million people were affected by this virus and it continues to be a major threat with a continual increase in cases around the world (World Health Organization 2021a). While countries like the United States of America, China, Germany, Australia, Brazil, Italy, and France are witnessing an alarming rise in the number of cases, a growing number of cases are also reported in Japan, India, and Portugal (World Health Organization 2021b).

The pandemic threatens to reverse decades of progress achieved worldwide towards eliminating child deaths that are preventable. Children with weak immune systems are generally prone to adverse effects, while children with healthy and stronger immune system may be spared of direct mortality effects of this infection. Reports of stillbirth during the COVID-19 pandemic due to fewer women having access to health care have increased stillbirths by 2,00,000 in the near term, which could have been avoided if proper antenatal and delivery care is implemented (UNICEF 2020).

As of March 2022, 3.7 million covid deaths were reported, including 0.4% of children and adolescents below 20 years of age. So far, the evidence does not point to an increase in child and youth mortality from COVID-19 until 2020, however these data have limitations of disproportional representation from high income and low income countries suspected of under reporting of regional variations. Women and children receiving essential health services worldwide have better child survival rate and a disruption in life-saving interventions can lead to more children dying of preventable diseases (Roberton et al. 2020; UNICEF 2021). A poorly developed immune system in children can make them susceptible to the attack of Covid 19. Functional foods (FF) are an alternative worth believed for the prevention of diseases and protection against COVID 19 in children. It has been established, over the course of more than two decades, that FF successfully boost both the cellular and humoral immune systems in children. Consumption of various foods that are high in probiotics and multivitamins can effectively prevent a variety of diseases (Angarita-Díaz et al. 2020; Haschke et al. 2001).

Components such as vitamins, minerals, carbohydrates, protein, and fat are major requirements that satisfy as the nutritional demands in children and adults food formulations. However, in children nutrient needs vary by age, for instance infants under the age of 6 months depends on breastfeeding, whereas 2-year-old require 1000 cal per day, while teens need 2400 cal per day that are to be met with food and additional supplements. WHO recommends 0.9 g/kg/day of protein for 3 to 18-year-old boys and 0.8 g/kg/day of protein for girls within the age of 3–18-year-old (Hörnell et al. 2013). Essential fatty acids, vitamins (A, C, D, E and B complex), minerals such as iron, calcium are some of the other nutritional components essential for children’s health to prevent against viral infections (Patel & Rouster 2021).

Prenatal and postnatal nutrition are important factors that majorly influences children’s gut microbiomes and metabolism. However, factors such as postnatal nutrition, composition of gut microbiota, its abundance, and their impact on immune system in children and in addition their role in non-communicable disease are poorly understood (Fragkou et al. 2021). FFs are rich in bioactive compounds that carry anti-viral and antimicrobial properties. However, these bioactive compounds present in FFs must be investigated for potential toxicological effects during diet formulation in children. The current review paper focuses on the potential utilization of FFs in combating COVID-19 infection in children. The palatability and safe limits of FFs and its components are extensively discussed. Further, the challenges in framing strong regulations for the use of FFs and its components is formulating dietary nutraceuticals for infant foods are highlighted in the review.

### Functional food components associated with its well-being in human

A food is considered “functional” if it can alter one or more target functions in the body in a manner that leads to either a better state of health and well-being or a reduction in the risk of illness (Plasek et al. 2020). Due to its constituents, functional food products can aid in the prevention of numerous chronic diseases (Alkhatib et al. 2017). Balanced food is composed of carbohydrates, proteins, fats, vitamins, and minerals for optimal health. Nutraceutical supplements containing those components are often misunderstood as functional foods (FF). FF are essentially fortified with specific non nutrient or dietary components that can elicit a physiological or therapeutic effect in humans upon consumption. These special components are majorly derived from plant-based foods, dairy foods, genetically engineered food and processed food. Functional foods provide nourishment to a wide range of bacteria which usually inhabits in the colon. In addition, functional foods produce metabolites that controls the level of cholesterol, improves the host immune system, provide necessary vitamins and minerals for the better metabolic functioning (Rani et al. 2018). The effect or the health claims of functional nutraceuticals are clinically traceable and are well characterised by suitable biomarkers or physiological changes (Alongi & Anese 2021; Iwatani & Yamamoto 2019). Utilization of FFs claimed to confer benefit in the reduction of serum triglyceride levels, hypertension, hypercholesterolemia, diabetes mellitus, bone health and other diseases (Iwatani & Yamamoto 2019). Some of the major components of functional foods and their proclaimed health benefits are listed in Table 1.

**Table 1:** Various functional ingredients used as functional food components for proclaimed health benefits

Proclaimed function	Active Component	References
GI tract health	Lactobacilli, Bifidobacterium, oligosaccharides, dietary fibres	(Walter 2008), (O’Callaghan & van Sinderen 2016), (Walsh et al. 2020),
Reduction of triglycerides	eicosapentaenoic acid, docosahexaenoic acid, dextrin, tea polyphenols	(Chahal et al. 2014; (Razdan & Pettersson 1994)
Cholesterol, LDL reduction	Chitosan, Chitin, Soy protein hydrolysate, Hesperetin	(Bokura & Kobayashi 2003), (Cho et al. 2007), (Kim et al. 2003)
Control of hypertension	Lactobacillus Helveticas derived Pro-Pro and Ile-Pro-Pro, γ-aminobutylic acid	(Bokura & Kobayashi 2003)
Antiviral property	Glycyrrhizin, Epigallocatechin gallate	(Cinatl et al. 2003), (Zhong et al. 2012)
Blood sugar level	Dextrin, guava polyphenol	(Sharma et al. 2022; Aliasgharzadeh et al. 2015; (Deguchi & Miyazaki 2010)
Tooth care	Palatinose, mannitol, erythritol, xylitol, tea polyphenol (prevent streptococcus mutants	(Gupta 2018) (Xu et al. 2011)
Mineral uptake	Casein phosphor peptide, calcium citrate malate	(Erba et al. 2002), (Reinwald et al. 2008)
Eye health	Lutein, blueberry anthocyanins	(Kizawa et al. 2021)
Memory and cognitive function	Ginko biloba, DHA, EPA	(Jackson et al. 2016)
Joint functions	Collagen hydrolysate, glucosamine	(Moskowitz 2000), (Zhu et al. 2018)

The worse quality of diets and the repeated shocks caused by the COVID-19 pandemic and its containment measures have led to an increase in malnutrition among the most vulnerable children in recent years. Disruption in food supply chain system during COVID 19 have affected the health and nutrition service. Children that are malnourished have compromised immune systems and are at a higher risk of dying from the COVID-19 virus. It may also be more challenging to provide the treatment and care that these children require (Fore et al. 2020). However, Inclusion of probiotics (for gut health), fermented dairy foods, essential fatty acids, dextrin, and tea polyphenols (for triglyceride reduction) and a balanced food, with essential supplementation will be vital in ensuring that the children’s immune system is protected during the pandemic. In addition to dietary supplements, life style adaptations towards consumption of functional foods (FF), fortified foods, breast feeding in infants can minimise the chances of acquiring communicable diseases (Chahal et al. 2014; Razdan & Pettersson 1994).

Various food components of natural origin are identified as immunomodulators, antiviral, antimicrobial, and anti-inflammatory agents that can be included in functional foods for children. Some of the widely known ingredients that can be included as component in functional foods are discussed below.

### Glycyrrhizin

**Fig. 1:**
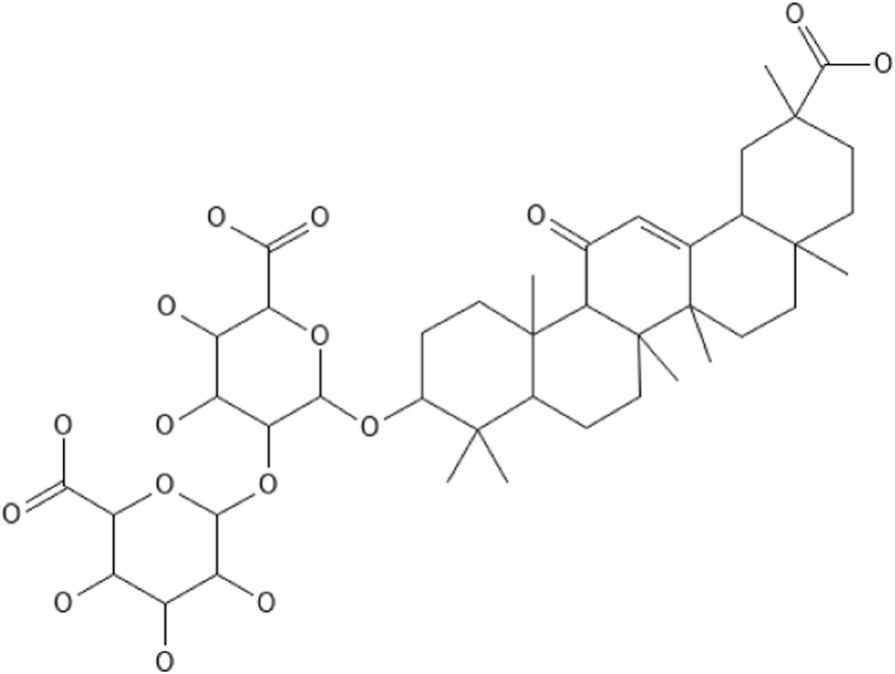
Structure of glycyrrhizin, a triterpenoid class of saponin found in liquorice

Glycyrrhizin is a triterpenoid saponin compound found in liquorice (Fig. 1). Clinical trials proved the inhibitory effect of glycyrrhizin against chronic hepatitis C and, HIV-1. Additionally, glycyrrhizin is effective against herpesvirus, epstein–barr virus, and influenza virus (Huan et al. 2021). With a selectivity index value of 67, glycyrrhizin is a potent inhibitor of SARS-CoV replication in the Vero cells that not only blocks viral replication but also limits the adsorption and penetration of viruses into new host. Compared to a standard (SARS strains FFM-1 and FFM-2 against Vero cells untreated with glycyrrhizin) virus adsorption, was less effective in glycyrrhizin supplemented group (EC50 600 mg/L vs 2400 mg/L) (Huan et al., 2021). According to studies, Disruption of lipid rafts that are required for covid viral entry in to cell is majorly affected by the GLR (glycoside of glycyrrhetic acid). With the advantage of GLR being categorized as GRAS component, it’s pharmacological activities on the host confers additional benefits such as antiviral and ant inflammatory properties. Further, GLR therapy (300 mg/ml) limited the replication of clinically isolates of COVID in Vero cells (Cinatl et al. 2003). Herbal formulations constituting GLR are widely used in treating respiratory infections (Yao et al. 2020). Similarly, the viral cell signalling pathways like casein kinase II, protein kinase C, and transcription factors such as nuclear factor κB and activator protein 1 are affected by glycyrrhizin. Furthermore, glycyrrhizin along with its aglycone metabolite 18β glycyrrhetinic acid upregulate the expression of inducible nitrous oxide synthase and the production of nitrous oxide in macrophages (Cinatl et al. 2003). Moreover, the inducible nitrous oxide synthase and the production of nitrous oxide in the macrophages are upregulated by 18β glycyrrhetinic acid, an aglycone metabolite of glycyrrhizin.

The safe limit for consumption of glycyrrhizin is limited to 100 mg per day in children of age 5–11 years. However, the total daily intake at 2 mg/kg/day is the safe range for glycyrrhizin administration in children around the age of 10 years (Tassinari et al. 2015). Overconsumption of glycyrrhizin (≥ 500 mg/week) could have caused adverse effects in mineralocorticoid related activity in 8 year old children (Kwon et al. 2020; Zeece 2020). Various side effects reported for higher intake of glycyrrhizin includes hypertension (van Beers et al. 2011; Wu et al. 1999), hypokalaemia (Meltem et al. 2009), hypertensive encephalopathy (Carfagnini et al. 2015; van Beers et al. 2011), rhabdomyolysis (Meltem et al. 2009; Shah et al. 2012) and cardiac arrest (Crean et al. 2009). Hence liquorice can be used in foods with caution for its health benefits.

### Epigallocatechin gallate (EGCG)

**Fig. 2:**
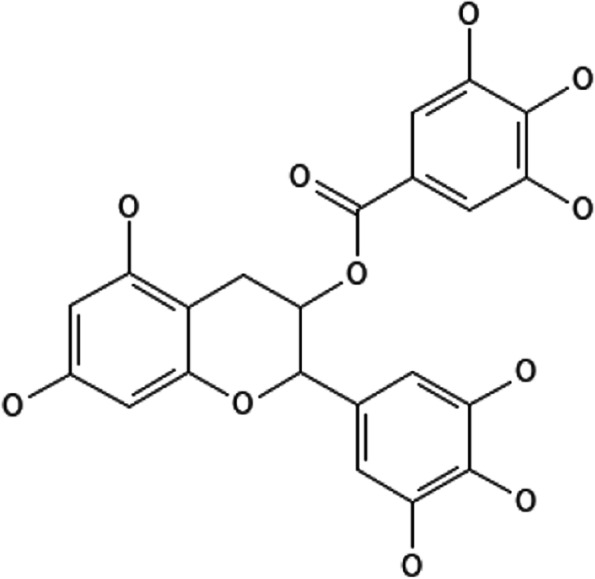
Structure of Epigallocatechin gallate

Epigallocatechin gallate (EGCG) is a plant-based catechin, which is better known as a primary active compound in green tea (Fig. 2). It also exists in foods such as cranberries, strawberries, kiwis, cherries, pears, apples, and in dry fruits like pistachios and hazelnuts at a minimal amount (Bhagwat et al. 2011). The catechin is further classified into a larger group of plant compounds called polyphenols. ECGC has been tested against several viruses for its antiviral activity and is considered as a potential therapeutic agent over synthetic drugs and can be used as an antiviral agent in the treatment of COVID-19. EGCG inhibited the spread of SARS-CoV-2, MERS, and SARS-CoV pseudo-typed lentiviral vectors resulting in reduced number of virus infections. Galloyle side chain of ECGC binds to the ACE2 receptor of SARS-CoV-2 virus and inhibit the interaction and binding with host receptors (Bimonte et al. 2021a). Molecular docking studies revealed better binding strength of ECGC compared to Hydroxychloroquine (HCQ) to the viral receptor. EGCG binds to all the three sites in the viral receptor ACE2, whereas HQC could bind only to one site of ACE2 receptor (Subbaiyan et al. 2020).

Mpro and Nsp5 is a hydrolase synthesized by coronavirus that aids in proteolysis at their mature stage. The Mpro can hydrolyse the original virus-encoded polyproteins (ppla, pplab) and 12 new non-structural proteins (essential and indispensable for virus survival) including RNA polymerase, helicase, and methyltransferase. Thus, making Mpro and Nsp5 an important molecule to target for the development of anti-COVID-19 drug development. Docking studies proved that out of the eight polyphenol compounds present in green tea, EGCG had highest binding affinity towards Mpro forming a stable Mpro-polyphenol complex inhibiting the replication of coronavirus (Wang et al. 2021). Similarly, RNA-dependent RNA polymerase (RdRp) can catalyze RNA replication and transportation of SARS-CoV-2. Molecular dynamic simulations revealed that EGCG can potentially bind to the active site of RdRp and cause suppression of RdRp expression in SARS-CoV-2 (Wang et al. 2021). The mechanism of action against Porcine Reproductive and Respiratory Syndrome Virus (PRRSV) is by inhibiting the virus replication, proliferation, and assembly. EGCG at 50 μM reduced the virus infectivity by 90% at an early stage of viral life cycle. In addition, usage of EGCG in children up to 800 mg per day is recommended to be safe to use in in-vitro studies (Younes et al. 2018). It is a wide-spectrum antiviral molecule that acts at various stages of the virus replication cycle (Bimonte et al. 2021b). ECGC also effectively inhibits the intermediate stages of the influenza virus life cycle by a hemagglutination inhibition (Song et al. 2005).

EGCG is considered to have a positive impact on health by reducing the inflammation rate. The antioxidant property of ECGC protects the cells from damage linked with oxidative stress, resulting in an anti-inflammatory effect (Ohishi et al. 2016). EGCG limits cellular damage caused by free radicals by acting as a potent antioxidant, whereas consuming functional foods high in catechin may help reduce the free radical damage (Kim et al. 2014). The presence of ECGC in green tea controls accumulation of cholesterol, reduces blood pressure, and associated risk factors that affect the cardiac system (Bhardwaj & Khanna 2013). Long-term consumption of green tea shows significant weight loss and helps to prevent degenerative brain disease (Jówko 2014). Effectiveness of liquified ECGC mouthwash in children (*n* = 47) belonging to age group between 5 to 12 revealed a significant reduction in the growth of mutant *Streptococci* and *Lactobacilli* found in the oral cavity of children. Thus, it acts as an antimicrobial agent in children by substituting the chlorhexidine-based mouth wash (Vilela et al. 2020).

### Allicin and diallyl trisulfide (DATS) from garlic

**Fig. 3:**
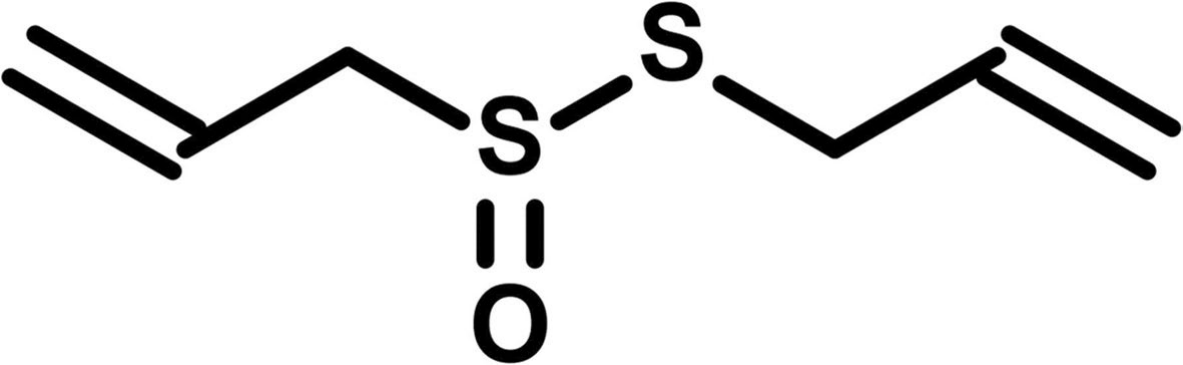
Structure of Allicin

Garlic a commonly used herb in medicinal formulations contains bioactive sulfur compound derivatives such as allicin, diallyl trisulfide which is an excellent source of vitamin C, vitamin E and polyphenols (Fig. 3). *Allium Sativum* could play a therapeutic role against COVID-19 by suppressing the proinflammatory cytokines and by regulating leptin expression through the modulation of immune cells. In innate immunity, leptin increases the cytotoxicity of natural killer (NK) cells and promotes the activation of granulocytes, macrophages and dendritic cells. Aged garlic extracts (AGE) are reported to supress proinflammatory cytokines such as TNF-α, IL-6, CRP, leptin receptor mRNA and PPAR-γ. AGE inhibit TLR signalling pathway and aids in the activation of AMPK, CD4+/CD8 T cells and NK cells (Mustafa & Orkide 2020). Antiviral properties of garlic against SARS-CoV and several other viruses such as herpes simplex virus (HSV- types 1 and 2), influenza B, and HIV are reported (Khubber et al. 2020). Garlic can be utilized in fresh or in dehydrated form where majorly the sulphur-containing phytochemicals in garlic contribute for anti-inflammatory, anti-cancer, anti-diabetic and cardioprotective properties (Batiha et al. 2020). In children, the safe and effective use of garlic derivatives provides protection against fungal infections. A daily intake of 3 g of garlic is considered to be safe to combat various respiratory and cardiovascular-related disorders (Davis 2005).

Allicin and diallyl trisulfide give garlic distillates their flavour with allicin being the most bioactive compound in garlic. The chopping of garlic cloves activates allicin and the non-proteinogenic amino acid known as alliin, which is the precursor of allicin (Sarvizadeh et al. 2021). In Silico studies found that allicin and DATS extracted from garlic can bind to structural protease of SARS-CoV-2 and thereby inhibit the replication process in the infected cells (Rajagopal et al. 2020).

### Fucoidan (sulfated polysaccharides) extracted from brown seaweeds

Fucan or fucosan, also known as “Fucoidan,” is a polysaccharide found in brown seaweed and in certain marine invertebrates, including sea cucumbers. In addition to sugars such as glucose, mannose, and galactose, brown seaweed fucoidans also contain galactose. Fucoidan consists of amino acids, vitamins, essential fatty acids, minerals etc. of high biological value (Catarino et al. 2018). Fucoidan is found to be effective against a variety of viruses such as Herpes Simplex Virus (HSV), SARS-CoV-2, and Human Immunodeficiency Virus (HIV) (Luthuli et al. 2019). Fucoidan from the blown algae binds and blocks the neuraminidase activity in Influenza A which inhibits the release of viruses (Helen Fitton et al. 2020).

The glycoprotein and ACE2 receptors of the host cells are utilized by SARS-CoV-2 for cellular entery. The mechanism of the action for viral entry takes place by modulating the viral spike protein with host cell ACE2 receptor (Yim et al. 2021). Fucoidan inhibitory effect for host cell infiltration were studied using pseudo-SARS-CoV-2 virus (Andrew & Jayaraman 2020; Kwon et al. 2020). The metabolites of seaweeds act as a potential agent to prevent influenza infection in children (Besednova et al. 2019). Fucoidan is also considered as a complementary medicine for managing diabetes mellitus in children (Cheng et al. 2019). Fucoidan is used to cure oedema, a symptom of kidney disease, and in addition, it reduces the inflammatory responses and improves glomerular filtration (Wang et al. 2019).

### Carrageenan

Carrageenan is a natural hydrophilic polysaccharide, extracted from red seaweeds. Different forms of carrageenan λ, κ, ι, ε and μ containing 22 to 35% of sulphate groups are reported in *Chondrus crispus*. Carrageenan is mainly used in food products such as chocolate milk, processed meats, etc. Carrageenan is effective against some enveloped viruses, including HSV-1, HSV-2, and swine fever viruses, and inhibits the encephalomyocarditis virus. Carrageenan is a vital support material for immobilising whole cells and is also used as semi-synthetic antibiotic effluent. In children infected with the common cold virus, the direct administration of carrageenan via nasal spray reduces symptoms of cold (Bodenteich et al. 2013). The polysaccharide inhibits the first stage of viral replication by attaching to the cell surface of the virus and prevents virus entry through its binding activity (Gonzalez et al. 1987; Varese et al. 2021). Carrageenan is used as nasal and mouth sprays that have the potential to act as a first line defense by inhibiting the infection and transmission of SARS-CoV-2. Studies by Fröba et al. (2021) demonstrated the antiviral effect of carrageenan against SARS-CoV-2 spike pseudotyped lentivirus particles (SSPL) and patient-isolated SARS-CoV-2 VOCs in A549ACE2/TMPRSS2 and Calu-3 human lung cells. The study revealed that iota-carrageenan could be effective in treatment of SARS-CoV-2 infections and shows hope to control future variant also.

### Galactan sulfate compounds

The source of sulfated galactan is red algae, and isolation of galactan is carried out from algae by extraction with water followed by the precipitation of polysaccharides with ethanol. Sulfated galactans can be an additive in sauces and ice creams, which can be served to children and can be considered as an alternative to gums (Waldron & Faulds 2007). Galactan sulfate is known to have therapeutic activities against infection caused by virus (Herpes Simplex Virus, Influenza virus and Human Immunodeficiency Virus type-1), bacteria, and fungus. In children, the galactan sulfate can be used as an anticoagulant (Patel 2012). The sulfated galactan is also reported to have a vital use as an antimicrobial and antiviral agent in children (Ahmadi et al. 2015; Pomin & Vitor. 2017).

The galactan sulphate suppresses the attachment of viral particles to the host cells by its anionic properties contributed by the presence of a high number of sulphate groups (Chattopadhyay et al. 2007). Simulation studies by Padmi et al. (2022) revealed that the sulfated polysaccharide (1- > 4)-beta-galactan has a binding energy of − 8.3 kcal/mol with ACE2 receptor. The result from molecular docking of (1- > 4)-beta-galactan in target protein ACE2 receptor had lowest binding energy reflecting better biological activity than the common SARS-CoV-2 drug such as remdesivir, molnupiravir, baricitinib, lopinavir, oseltamivir, and favipiravir. Macroalgae compounds are predicted to have potential antiviral activity against SARS-CoV2. However, an extensive study to investigate therapeutic potential of sulfated galactan’s as inhibitory substance in combating SARS-CoV-2 in in-vivo systems are yet to be deduced.

### Fermented foods and their role as functional foods

Fermented foods are foods or beverage products derived through the process such as controlled microbial growth interventions and the conversion of food components through various enzymatic action. *Lactobacillus* and *Bifidobacterium* are considered as the most used bacterial types in the process of fermentation in food product such as yoghurt, kefir, kimchi, miso, tempeh and kombucha (Tamang et al. 2020). Fermented foods possess antiviral property by increasing the phagocytic activity in the host tissues and helps in the production of immunoglobulin mediated defense by the process of elimination of intracellular pathogens. Few of the beneficial bacteria and their role in preparation of functional foods are illustrated in Table 2. Fermented foods also help in promoting the microbiota modulation and influence the metabolic pathway, especially associated with metabolism of dietary components and some host-generated substances. Fermented functional food components are active against SARS-CoV-2 and the mechanism of action is not direct but through the effective stimulation of the body’s immune system, which enhances natural killer cell toxicity, secretion of pro-inflammatory cytokines and by enhancing the production of T lymphocyte cytokines (CD3+, CD16+, CD56+) (Gill & Guarner 2004; Muhialdin et al. 2021). Fermented foods are found to be effective in enhancing the function of the gut microbiota, promoting the mucosal immunity, and by acting as an antiviral agent in respiratory infections (Kesika et al. 2022).

### Probiotics associated with functional foods

**Fig. 4:**
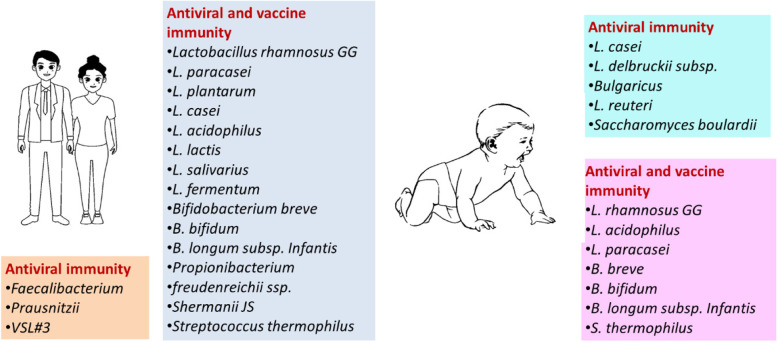
Probiotic that possesses antiviral properties as demonstrated in adults and children

Probiotics are a combination of microbes found in yoghurt, kefir, pickles, fermented fruits, vegetables. Reports of probiotic drinks displaying therapeutic properties fighting against SARS-CoV-2 are elucidated (Nasri et al. 2014). Currently, the exact mechanisms of probiotics’ antiviral properties against COVID 19 are unknown. In an experimental model of respiratory infection, a precise Th1 balance favoring the assembling of IgG instead of IgE and rising levels of interleukin-10 and IFN-y cytokines, was observed in the host which controls the severity of viral infection (Vlasova et al. 2019). Certain probiotics that possess antiviral properties in children and adults are depicted in Fig. 4. Antiviral activity is summarised by enhancing the mucosal innate immune system by altering the acquired immune response through regulatory and other anti-inflammatory responses (Infusino et al. 2020).

Few clinical studies have been criticised for not providing enough information regarding probiotic strain(s) employed in the making of functional food as health benefits often depend on the strain. The probiotic strain may lead to prophylactic and immune-boosting effects in managing COVID-19 and treating respiratory infections (García-Burgos et al. 2020). Probiotics are found to be effective against self-reported symptoms of upper respiratory tract infection in children, thus, probiotic bacteria can be considered as an effective agent against viral particles (King et al. 2014). Function of the respiratory microbiome is influenced by the interaction between the gut-lung axis. An essential pathway known as the mesenteric lymphatic system acts as a link between lungs and the intestine. The metabolites or fragments released by gut bacteria are translocated across the intestinal barriers reaching the systemic circulation and contribute in the modulation of lung immune response (Enaud et al. 2020). Supplementation of probiotic food in infant food formula mimics the effect of bacterial community present in breast milk and exhibit similar synergistic effect. Anti-infective effects are reported in various studies by the bacterial community present in human breast milk. Binding of the probiotic’s strains present in the infant foods strengthens the intestinal barrier function due to higher epithelial adhesion and better systemic immunostimulation. Probiotic strains such as *Lactobacillus fermentum*, *Saccharomyces boulardii* and *Lactobacillus rhamnosus* in infant foods are very well tolerated (Maldonado 2019). New born with less birth weight showed progressive adaptation in gut stability when supplemented with *Bifidobacterium lactis* (Chi et al. 2019). Incidence of respiratory infection reduced drastically due to supplementation of *Lactobacillus fermentum* CECT 5716 in infant formulations (Maldonado et al. 2012).

### Food additives as nutraceuticals

A nontoxic, glycoprotein named Lactoferrin binds to cell surface of viral molecules, slowing the HIV replication and prevent the viral entry into the cells. Lactoferrin activates T-cells, suppresses IL-6 and TNF-α, and downregulates ferritin. In umbilical vein cells lactoferrin reduced the oxidative stress indued by hydrogen peroxide. Lactoferrins are non-toxic glycoprotein that can inhibit viral entry via binding to both cell surface as well as to the viral particles. Therefore, lactoferrin could play a crucial role in preventing the entry of SARS CoV virus and its replication in infants. A clinical study carried out using zinc saturated lactoferrin in infant formulas at a dose of 100 mg to 4.5 g per day enabled the COVID-19 prevention and treatment (Mrityunjaya et al. 2020).

Piperine obtained from pepper possess anti-inflammatory and antiviral properties that can be used to reduce COVID-19-induced inflammation. Piperine inhibits IL-6 and MMP-13 to reduce PGEs. Piperine promote activity of phagocytes to support immunity, and inhibits mRNA expressions of LPS-induced IRF-1, IRF-7, IRF-3 phosphorylation, STAT-1 activity, and type 1 IFN expression. In rat models piperine deficiency results in elevated oxidative stress characterized by uncontrolled pro-inflammatory TNF- and VCAM-1 expression. Supplementation of zinc can partially reverse the lung tissue remodeling caused by the inflammation. Cinnamaldehyde is a bioactive compound with potential anti-inflammatory activity that could be used in the mitigation of SARS-Cov-2 induced hyper inflammation in the lungs. Cinnamaldehyde suppresses TNF induced inflammation by inhibiting NF-B, endotoxin-mediated inflammasomes such as NLRP3, TLR4 and NOD (Mrityunjaya et al. 2020).

### Palatability considerations of certain compounds present in FFs

The sensory characteristic of food influences the likeness of food in children. The center of influencing the control of energy intake within and across meals is palatability (Mccrickerd & Forde 2016). Glycyrrhizin is commonly employed as a sweetener in cold beverages and in pharmaceutical preparations such as haematic pills. It is 50–100 times sweeter than sucrose, with slower onset of sweetness. Therefore, it helps to mask the bitter flavour of other infant preparations. The herb’s sweet, moist, and calming qualities aid in detoxification and act as an anti-inflammatory agent. After drying, powdering, and other processing, liquorice root extract is used in infant formulas. The infant formula contains EGCG to inhibit the growth of *Bacillus cereus*, specifically in infant rice cereal (Cetin-Karaca & Newman 2017). Primarily bitter and astringent, the palatability of EGCG can be improved by increasing the concentration Na-caseinate (Narukawa et al. 2010). Allicin (diallyl thiosulfate) is the most common bioactive constituent of garlic that is responsible for its distinctive pungent odor in baby foods (Suleria et al. 2015).

Fucoidan is a polysaccharide largely made up of l-fucose and sulfate groups that provides therapeutic effects that make it favorable among food and pharmaceutical industry; however, it has a distinct nutritional value in infant formula by acting as an anticancer agent and immunoregulator. It is utilized as liquid formulated nutraceutical product which provide various health benefits. Fucoidan is unique because it has no taste or odour (Qin 2018). Carrageenan is primarily employed as a gelling and thickening agent. Fucoidan is primarily present in infant formula, yoghurt, cheese, and ice cream (Liu et al. 2021). Due to their gelling and thickening properties in infant formula, galactan sulphate is widely used in the food industry and is of great commercial importance. It is odourless and tasteless with a mucilaginous consistency that would not affect the palatability of infant foods (Vavilala & D’souza 2015).

### Toxicological aspects of functional food components

While discussing the therapeutic benefits of these natural compounds it is noteworthy that their toxicological and allergic potentials are important to be examined. Table 3. provides an insight to the various toxicological aspects of the compounds discussed in this review. Oral consumption of 10 mg/day of glycyrrhizin is considered safe in around 8-year-old children. An average daily intake of liquorice dried roots should not exceed more than 5–15 g as it accumulates glycyrrhizin equivalent to 200–600 mg in children. Over consumption of glycyrrhizin causes a condition called pseudo aldosteronism, which can cause a person to become overly sensitive to a hormone in the adrenal cortex. This condition can lead to headaches, fatigue, high blood pressure, and even heart attacks. Therefore, the use of glycyrrhizin for infants is not recommended without the instruction of a pediatric doctor (Isbrucker & Burdock 2006a; Omar et al. 2012; Räikkönen et al. 2010).

**Table 2:** List of Microbes used in functional foods and its benefits

Microbes	Sources of functional food	Function	References
*Lactobacillus buchneri, Lactococcus lactis subsp. Lactis*	White Liquor	Used as a bacterial inoculant to improve the aerobic stability of foodstuff.	(Xiu et al. 2012)
*P. pentosaceus, W. cibaria, W. paramesenteroides*	Suan-tsai, fu-tsai	Used as a starter culture for fermented foods such as various meats, vegetables.	(Chao et al. 2008)
*Enterococcus thailandicus, Pediococcus ethanolidurans*	Chinese pickled vegetables, chesses, yoghurts	Used as a silage inoculant in the creation of sausages, cheeses etc.	(Jie et al. 2012a)
*L. plantarum, Weissella cibaria*	Douchi - Fermented and salted black soyabean	Used in a mixed starter culture for making breads.	(Liu et al. 2012)
*E. faecalis, Enterococcus durans*	Fermented yak, mare, goat, and cow milk products	Used as a commensal bacterium inhabiting the gastrointestinal tracts of humans.	(Jie et al. 2012b)

**Table 3:** Toxicological and dose considerations of functional food components

Name	Toxicological aspect	Reference
Glycyrrhizin	Non-mutagenic, non-teratogenic, anti-genotoxic properties. Actively inhibits 11 beta-hydroxysteroid dehydrogenase type 2, the fetoplacental barrier that provides greater cortisol levels in children and thus associates with HPAA function. Safe oral dose: 10 mg or less	(Isbrucker & Burdock 2006b; Omar et al. 2012; Räikkönen et al. 2010)
EGCG	High antioxidant property, Pro-oxidative properties at larger doses. Associated with hepatotoxicity at higher doses Safe oral dose: 800 mg/ day or less	(Sergi 2020a, 2020b)
Allicin and Diallyl Trisulfide	Cause allergic reactions and toxicity in normal infant body cells. Diallyl sulphate is a selective inhibitor of cytochrome P450 2E1 and inhibits the metabolism of xenobiotics such as analgesic drugs. A dose of 1 mg or less of allicin twice daily is considered safe.	(Ansary et al. 2020; Rao et al. 2015)
Fucoidan	No oral toxicity is observed. Plays a vital role in preventing neurotoxic effects caused by beta-amyloid A daily dose of 4 g seaweed for up to two months is considered safe.	(Feldman et al. 1999; Fitton 2011)
Carrageenan	Toxicological properties at higher doses Non carcinogenic It is not absorbed by the GI tract and has no immune system effects in children. A daily dose of less than 75 mg per kg body weight is considered safe.	(Cohen & Ito 2002; Younes et al. 2018)
Galactan sulfate	Moderate toxicological effects and immunostimulant effect in children Safe oral dose: A daily dose of less than 1200 mg	(McKim et al. 2019)
Probiotic functional foods	Can be deadly in large quantities with detectable toxicological consequences. The daily intake of probiotic-containing dairy products is considered an appropriate way to ingest probiotic bacteria in infants.	(Žuntar et al. 2020) (Twetman & Stecksén-Blicks 2008)
Fermented functional foods	Toxicological concerns on the ethanol-containing fermented foods The acute toxicity in children at a dose level exerted by ethanol is 0.3 g/kg of body weight.	(Gorgus et al. 2016)

Epigallocatechin gallate (EGCG), a unique compound isolated from plant is known for its potential positive impact on health by reducing inflammations in heart, liver, and brain diseases. However, over dosage in infants (maximum of 16.50 g/day), toddlers (minimum of 7.31 g/day and maximum of 23.75 g/day), and children (minimum of 16.67 g/day and maximum of 125.65 g/day) of EGCG causes pro-oxidative properties that could trigger nuclear factor erythroid related factor 2 (NRF2) that regulate regulating cellular antioxidant responses. Excessive consumption has been linked to hepatotoxicity and hepatocellular damage leading to subcellular and cellular damage in infants and children (Sergi 2020a, 2020b).

Diallyl trisulphide (DATS) is beneficial in reducing serum cholesterol in patients suffering from hyperlipidemic disorder, cancer, diabetes/hyperlipidemia, HIV, Parkinson’s disease. DATS are metabolized rapidly inside body to form various derivative products such as diallyl sulfide (DAS), diallyl sulfoxide (DASO), diallyl sulfone (DASO_2_) and allyl mercaptan. The allyl mercaptan is a breakdown product of DAS.DAS, DASO, DASO_2_ by S-oxidation. Further these allyl mercaptan are converted to epoxides and GSH conjugates prior to elimination from the body. The epoxides formed during S-oxidation are toxic to hepatic as well as extra-hepatic cells and can cause autocatalytic destruction of hepatic enzymes such as Alkaline phosphatase (ALP), Alanine transaminase (ALT) and Aspartate transaminase (AST). Children can safely consume 1 mg of allicin per day that helps in reducing advanced gastric lesions, cholesterol levels and oxidative stress. Overdosage in children could lead to oxidative stress in erythrocytes and DNA damage in lymphocytes (Ansary et al. 2020; Rao et al. 2015).

Sulfated polysaccharides such as fucoidans extracted from brown seaweeds have wide spectrum of biological activity such as anticoagulant, antithrombotic activity, and possess antiproliferative effect against viral infections. No toxicity effects of fucoidans from different brown algae are known due to limited number of studies. However, toxicological studies of fucoidan extracted from Okinawa mozuku (*Cladosiphon okamuranus*) in reference to varying levels of concentration in Wister rats after oral consumption demonstrated no toxicological effects at a dosage of 600 mg/kg per day. Even so, fucoidans at concentrations above 1200 mg/kg per day the blood clotting time in Wister rats were found to be prolonged significantly. Infants can safely consume 4 g of seaweed per day for 2 months (Feldman et al. 1999; Fitton 2011).

Carrageenan derived from red seaweeds are commonly used as food additives to contribute for textural stability in infant food products. Food grade carrageenan have higher molecular weight greater than 100,000 Da and thus may not degrade in gastrointestinal tract causing absorption issues in the system. Nevertheless, a substance known as poligeenan (degraded form of carrageenan) exhibits toxicological effects at high dosage. Since poligeenan can be toxic, stability and storage of carrageenan should be taken care before administration in adults and infant foods. Consumption of less than 75 mg of carrageenan per kg of infant body weight is considered to be safe (Cohen & Ito 2002; Younes et al. 2018). Galactan has moderate toxicological effects on children and activates toxicological macrophages. Children can safely take 1200 mg of *Coriolus versicolor* dry extract per day for 2 weeks and over dosage of galactan can cause inflammations (McKim et al. 2019).

Safety and toxicological risk of probiotic FF can be viewed two ways. The first involves adverse effects of probiotics and the second factor is related to undefined quality standards. The only standardisation of probiotic safety evaluation is a retrospective epidemiologic study and an in-depth pharmacological and toxicological post-marketing study (Twetman & Stecksén-Blicks 2008). In a clinical trial conducted for bacteremia (viable bacteria in the blood) in all the *Lactobacillus* strains, the presence of *Lactobacillus rhamnosus* in blood was reported in two separated cases. One of these cases was an infant who had received probiotic through gastrostomy tubes, and the other one was a 6-year-old child. Also, there are case reports of overt sepsis due to bacteremia accompanied by three main strains of *Lactobacillus* (*acidophilus*, *casei* and GG) followed by *Saccharomyces boulardii*, *Bacillus subtilis* and *Bifidobacterium breve*. Fungemia is another adverse effect caused due to the use of probiotics such as *Saccharomyces boulardii* and *Saccharomyces cerevisiae* that were found in the blood cultures of patients. Fungemia was identified in some critically ill patients who were administered probiotic such as *Saccharomyces boulardii* in order to stop antibiotic-dependent diarrhea (Sotoudegan et al. 2019). Reports of gastrointestinal side effects such as vomiting, nausea, spasms, diarrhea, bloating, thirst, and taste disturbance after use of probiotics in adults and children have been reported previously (Goldenberg et al. 2017).

The fermented food cause toxicological concerns related to the exposure of infants in both short-term and long-term use of ethanol-containing fermented FF, which are marketed on a non-prescription base. There is limited acute or chronic ethanol toxicity data for children, and acute toxicity for infants and young children with more than 50 mg/dL are in the risk of profound hypoglycemia and requires immediate emergency assistance (Gorgus et al. 2016).

### Regulations on infant food around the world

The focus of food science and technology has shifted from the previous goal of improving food safety and flavour to providing healthy functional attributes in disease management. In order to achieve industry development and commercialization strategies, there is a need for innovation in formulation of functional food (Ghosh 2012). Therefore, innovation in functional foods will collaborate the ideas along with the appropriate guidance from experts in food science, regulation, and management. Functional foods were non-commercialized food product freshly prepared from raw materials available in once household hence there was no necessity in framing regulations to define these products (Thompson & Moughan 2014).

In the late 90’s, research in functional foods were promising as ingredients in food formula were introduced to enhance food quality and function. Thus, urgent call for legislation in the field of infant functional food formula was not required (Hasler 2002). Later, the United States authority known as the Federal Foods & Drug and Cosmetic Act (FFDCA) had set forth regulation for formulating infant functional food to regulate the misuse of additives that could affect the health of infants and children (Ross 2000).

The European Commission of 2000, Food Safety had proposed around 80 new and authorized legislation in the field of regulating functional foods by establishment of general food laws, and creating an independent authority called Food Authority, that has brought up certain scientific advice on risk management on functional foods in infants (Coppens et al. 2006). Thus, the European Commission established the Regulation No. 1881/ (2006) limiting the nitrates and heavy metals in infant foods (Hardisson et al. 1996).

Consumption of vegetables, fish, meat and dairy products contaminated with nitrates and nitrites on a daily basis is a threat to infants of age below 12 months (Cortesi et al. 2015). The committee that evaluated the risks associated with the consumption of nitrite in southern Italy Joint suggests that the consumption of nitrites by infants leads to risk greater than Acceptable daily intake (ADI) (Gangolli et al. 1994). The Govt. of India put forth the Convention of Rights of the Child, and framed regulations with regards to the formulation of infant foods, following the declaration of act Rights of the Child in 1990. The meetings called attention to the major use of additives in infant foods in India and regulating the use of harmful additives (Das 2007).

The Association for Consumer Action on Safety and Health (ACASH) authorized by government regulates food violation policy. The infant milk substitutes, feeding bottles, and infant foods act, 1992 introduced the regulation of manufacture, supply and evaluates the distribution of infant milk and its substitutes (Das 2007). Recently, the World Health Organization had brought up a strategy across the globe to counter “diet-related non-communicable diseases (WHO 2002). There are numerous obstacles in introducing functional foods into the mainstream. The challenges are spread across several areas, ranging from functional food development to regulations. Functional foods contain a wide range of active ingredients that makes it difficult to determine the safety of these components, especially in children (Spence 2006). It is critical to create a proper regulatory mechanism for monitoring functional foods. There is a fine line between supplements and functional foods, making it difficult for regulations to distinguish between them (Storey 2004). Furthermore, because functional foods are made up of multiple ingredients, determining the nutritional composition requires cutting-edge analytical techniques (García-Cañas et al. 2012). Among these challenges, the development of functional foods opens a massive market opportunity in the event of a life-threatening pandemic. Improved health, lower healthcare costs, and bolstered rural economies are all attributed to the development of functional foods and nutraceutical products.

## Conclusion

FF are becoming more important in maintaining a healthy lifestyle. Pandemics such as Covid 19 highlighted the importance of consuming a diet rich in all essential nutrients. A lack of nutrients can make children vulnerable to disease. Multiple covid 19 variants and other viruses endanger public health. Children can only be effectively protected from such dangers if they are properly fed. Incorporating nutraceutical ingredients into new functional food formulations can boost the immune system and prevent disease. In this difficult situation, the inclusion of such antiviral components has the potential to slow the spread of the pandemic. Demand for functional foods is on the rise, particularly in developed economies where both health consciousness and disposable income are rising. Better regulations and newer technologies will foster the development of functional food for children which require additional amount of safety.

## Data Availability

The datasets used and analysed during the present study are available from the corresponding author on reasonable request.

## Abbreviations

SARS-CoV-2: Severe Acute Respiratory Syndrome Coronavirus 2
COVID-19: Coronavirus Disease 2019
FF: Functional Foods
ECGC: Epigallocatechin gallate
HCV: Hepatitis C virus
HIV: Human immunodeficiency virus
HSV: Herpes simplex virus
ACE2: Angiotensin-converting enzyme2
FAO: Food and Agriculture Organization
WHO: World Health Organization
LDL: Low density lipoprotein
GI Tract: Gastrointestinal Tract
IgG: Immunoglobulin G
IgE: Immunoglobulin E
CD3+/4+/6 +: Cluster of differentiation 3+/4+/6+, +)
TNF: Tumour Necrosis Factor
IRF: Interferon Regulatory factor
STAT: Signal Transduced and Activator of Transcription
IFN: Innate Interferon
VCAM: Vascular Cell Adhesion Molecule
TLR: Toll Like Receptor
HPAA: Hypothalamic Pituitary Adrenocortical Axis
